# Accurate Image Reconstruction in Dual-Energy CT with Limited-Angular-Range Data Using a Two-Step Method

**DOI:** 10.3390/bioengineering9120775

**Published:** 2022-12-06

**Authors:** Buxin Chen, Zheng Zhang, Dan Xia, Emil Y. Sidky, Taly Gilat-Schmidt, Xiaochuan Pan

**Affiliations:** 1 Department of Radiology, The University of Chicago, Chicago, IL 60637, USA; 2 Department of Biomedical Engineering, Marquette University and Medical College of Wisconsin, Milwaukee, WI 53201, USA; 3 Department of Radiation and Cellular Oncology, The University of Chicago, Chicago, IL 60637, USA

**Keywords:** dual-energy CT, two-step method, limited-angular range, directional total variation

## Abstract

Dual-energy CT (DECT) with scans over limited-angular ranges (LARs) may allow reductions in scan time and radiation dose and avoidance of possible collision between the moving parts of a scanner and the imaged object. The beam-hardening (BH) and LAR effects are two sources of image artifacts in DECT with LAR data. In this work, we investigate a two-step method to correct for both BH and LAR artifacts in order to yield accurate image reconstruction in DECT with LAR data. From low- and high-kVp LAR data in DECT, we first use a data-domain decomposition (DDD) algorithm to obtain LAR basis data with the non-linear BH effect corrected for. We then develop and tailor a directional-total-variation (DTV) algorithm to reconstruct from the LAR basis data obtained basis images with the LAR effect compensated for. Finally, using the basis images reconstructed, we create virtual monochromatic images (VMIs), and estimate physical quantities such as iodine concentrations and effective atomic numbers within the object imaged. We conduct numerical studies using two digital phantoms of different complexity levels and types of structures. LAR data of low- and high-kVp are generated from the phantoms over both single-arc (SA) and two-orthogonal-arc (TOA) LARs ranging from $14^{\circ }$ to $180^{\circ }$. Visual inspection and quantitative assessment of VMIs obtained reveal that the two-step method proposed can yield VMIs in which both BH and LAR artifacts are reduced, and estimation accuracy of physical quantities is improved. In addition, concerning SA and TOA scans with the same total LAR, the latter is shown to yield more accurate images and physical quantity estimations than the former. We investigate a two-step method that combines the DDD and DTV algorithms to correct for both BH and LAR artifacts in image reconstruction, yielding accurate VMIs and estimations of physical quantities, from low- and high-kVp LAR data in DECT. The results and knowledge acquired in the work on accurate image reconstruction in LAR DECT may give rise to further understanding and insights into the practical design of LAR scan configurations and reconstruction procedures for DECT applications.

## 1. Introduction

Dual-energy computed tomography (DECT) has found applications in clinical and industrial settings. In current DECT, one generally acquires data of low- and high-kVp X-ray spectra over a full-angular range (FAR) of 2π, or over at least a short-scan angular range [1,2,3,4]. Interest remains in the development of DECT imaging over limited-angular ranges (LARs) that are considerably less than the FAR of 2π (or than the short-scan angular range,) because such LAR scans may bear implications for radiation dose reduction, scan time minimization, and collision avoidance between the scanner and the imaged object. Inspired by the directional-total-variation (DTV) work on image reconstruction from LAR data in conventional single-energy CT (SECT) [5], we have investigated image reconstruction previously from LAR data in DECT [6,7] by focusing on the correction only for LAR artifacts and using DTV constraints in the reconstruction of kVp images followed by an image-domain decomposition. Other methods have also been developed for DECT with LAR data, but the angular ranges are generally not smaller than $90^{\circ }$ [8,9].

In this work, we propose a two-step method to reconstruct quantitatively accurate images in DECT from LAR data by correcting for both BH and LAR artifacts, thus improving the quantitative accuracy of images reconstructed and physical quantities estimated. In the method, from LAR data of low- and high-kVp, a data-domain decomposition (DDD) algorithm [10] is used first for obtaining LAR basis data in which the BH artifacts are compensated for; and a DTV algorithm [5] is then developed and tailored to reconstruct basis images from LAR basis data obtained. The reconstructed basis images can be combined to form virtual monochromatic images (VMIs), i.e., the X-ray linear attenuation coefficients, for visual inspection, and can be used also for estimating physical quantities such as iodine-contrast concentrations and effective atomic numbers within the imaged object [11,12,13,14]. We hypothesize that images and physical quantities with both BH and LAR artifacts corrected for in LAR DECT are quantitatively comparable with those obtained in FAR DECT. Therefore, in this work the results obtained for LAR DECT are compared with those obtained from FAR data in DECT in which BH artifacts are corrected for by using the DDD algorithm.

Numerical studies are conducted with a chest phantom [15] and a suitcase phantom [6] containing distinct anatomies and structures of potential relevance in medical and security applications [11,16,17,18,19]. Low- and high-kVp data are collected with single-arc (SA) or two-orthogonal-arc (TOA) scans of LAR [6], ranging from $14^{\circ }$ to $180^{\circ }$. Using the DDD and DTV algorithms, we estimate basis data and then reconstruct basis images, followed by the formation of VMIs at energies of interest from the basis images reconstructed. In addition to visual inspection and quantitative analysis of VMIs obtained, we also estimate iodine-contrast concentrations in chest images and effective atomic numbers in suitcase images from data of different LARs. Furthermore, we investigate image reconstructions from data acquired with SA and TOA scans of possible implications for potential non-diagnostic imaging applications involving, e.g., C-arm DECT, in which workflow or safety concerns may limit the scan angular range. The two-step method and the study design in the work can also be applied to investigations concerning image reconstruction in DECT and multi-spectral CT using techniques with sandwiched detectors [2], sequential scans [20], or advanced photon-counting detectors [21,22]. DECT with fast-kVp-switching X-ray tubes can also collect approximately overlapping rays [4].

## 2. Materials and Methods

### 2.1. Scans of Limited-Angular Ranges

**Figure 1:**
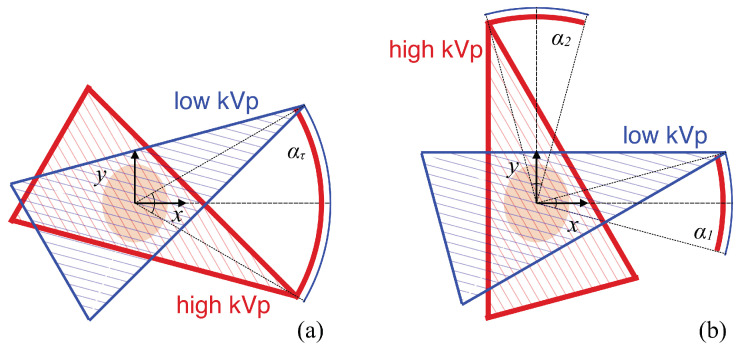
Schematics of SA (**a**) and TOA (**b**) scans of LARs in fan-beam DECT. The SA scan includes a pair of completely overlapping arcs of LAR $\alpha_{\tau }$, and the *x*-axis intersects with the middle point of the two arcs, whereas the TOA scan includes two pairs of completely overlapping arcs of LARs $\alpha_{1}$ and $\alpha_{2}$, and the *x*- and *y*-axis intersect with the middle points of the two pairs of arcs. For each pair of the completely overlapping arcs in the SA and TOA scans, low- and high-kVp data are collected over one of the paired arcs. In this work, we consider $\alpha_{1}=\alpha_{2}=0.5\alpha_{\tau }$.

In this work, we consider single-arc (SA) or two-orthogonal-arc (TOA) scans in a fan-beam DECT, as shown schematically in Figure 1a,b. The SA scan includes a pair of completely overlapping arcs of LAR $\alpha_{\tau }$, whereas the TOA scan includes two pairs of completely overlapping arcs of LARs $\alpha_{1}$ and $\alpha_{2}$. For each pair of the completely overlapping arcs in the SA and TOA scans, low- and high-kVp data are collected over one of the paired arcs. In this work, we assume that the *x*- or *y*-axis intersects with the middle point of each pair of the completely overlapping arcs, and that the tangential directions at the middle points of the two pairs of completely overlapping arcs in the TOA scan are orthogonal with each other in Figure 1b. We use $\alpha_{\tau }$ to denote the LAR of an SA scan and investigate image reconstruction from data collected over SAs of LARs $\alpha_{\tau }=14^{\circ },20^{\circ },30^{\circ },45^{\circ },60^{\circ },90^{\circ },120^{\circ }$, $150^{\circ }$, and $180^{\circ }$. For an SA of LAR $\alpha_{\tau }$, we also consider a TOA scan with two arcs of equal LARs satisfying $\alpha_{1}=\alpha_{2}=0.5\alpha_{\tau }$. (The work can readily be generalized to a TOA scan with two arcs of different LARs [23]).

**Figure 2:**
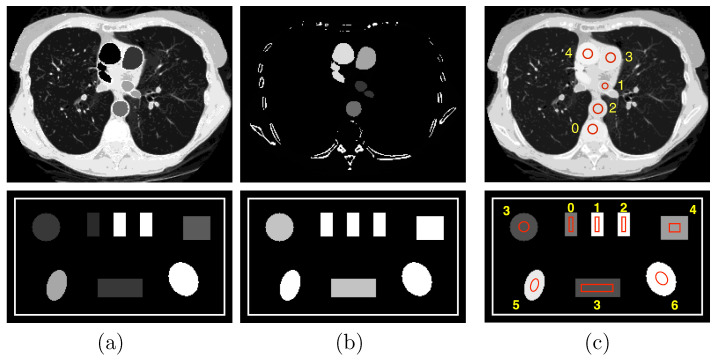
Row 1: (**a**) water and (**b**) iodine basis images and (**c**) VMI at 100 keV of the chest phantom; and row 2: (**a**) photoelectric effect (PE) and (**b**) Compton scattering (KN) basis images and (**c**) VMI at 40 keV of the suitcase phantom. Display windows for the chest phantom are [0, 1.2] for the two basis images and [0, 0.22] cm${}^{-1}$ for the VMI, while those for the suitcase phantom are [0, 0.22] and [0.1, 0.65] cm${}^{-1}$, respectively.

Dual-energy data are generated from a chest phantom and a suitcase phantom in Figure 2 with two different fan-beam geometries used in the numerical study: for the chest phantom, the source-to-rotation distance (SRD) and source-to-detector distance (SDD) are 100 cm and 150 cm, with a linear detector of 70 cm comprising 896 bins, whereas for the suitcase phantom, the SRD and SDD are 100 cm and 150 cm, with a linear detector of 32 cm including 512 bins. The imaged objects are assumed to be completely within the field-of-view of the scan configurations, resulting in no truncation. In the studies involving both phantoms, the angular interval is fixed at $0.25^{\circ }$ between two adjacent views. Dual-energy data are also collected over two full rotations, or the FAR of $360^{\circ }$, and images reconstructed from FAR data may be used as references in the work.

### 2.2. Imaging Model

In DECT, data are collected at ray *j* with two distinct spectra, referred to as low- and high-kVp spectra, and an imaging model can be expressed as [8]

(1) $$\begin{array}{c} \begin{array}{cc} g_{j}^{L} & =-\ln\sum_{m}^{M}q_{jm}^{L}\exp\left(-\sum_{i}^{I}a_{ji}f_{mi}\right),\\ g_{j}^{H} & =-\ln\sum_{m}^{M}q_{jm}^{H}\exp\left(-\sum_{i}^{I}a_{ji}f_{mi}\right), \end{array} \end{array}$$

where $g_{j}^{L}$ and $g_{j}^{H}$ denote model data of the low- and high-kVp scans; $q_{jm}^{L}$ and $q_{jm}^{H}$ the low- and high-kVp spectra after normalization (including possibly filtered tube spectra and detector response) at energy bin *m*; $a_{ji}$ the contribution of image pixel *i* to data of ray *j*; and $f_{mi}$ the image value at pixel *i* within energy bin *m* of the monochromatic image, i.e., the linear attenuation coefficient.

In the absence of the basis-decomposition error, $f_{mi}$ can be written as the combination of two basis images, i.e.,

(2) $$\begin{array}{c} f_{mi}=\mu_{0m}b_{0i}+\mu_{1m}b_{1i}, \end{array}$$

where $b_{ki}$ denotes basis image *k* at pixel *i* and $\mu_{km}$ the linear attenuation coefficient at energy bin *m* for basis material *k* (k=0 or 1). Image $f_{m}$, with $f_{mi}$ as its elements, obtained with Equation (2) is referred to also as the virtual monochromatic image (VMI).

In the work, assuming $\mu_{km}$, $q_{jm}^{L}$, and $q_{jm}^{H}$ are known, the two-step method is proposed for accurately reconstructing basis images $b_{ki}$, or, equivalently, VMI $f_{mi}$, from data collected over an SA or TOA of LARs in fan-beam DECT.

### 2.3. Numerical Phantoms Studied

We consider in the work two phantoms, i.e., the chest phantom [15] and suitcase phantom [6] shown in Figure 2, motivated by their possible implications in medical and security imaging, two distinct DECT imaging applications, and their distinctly different anatomic structures for evaluating algorithm performance. The chest phantom contains four regions of interest (ROIs) 1–4 with iodine-contrast agents at concentrations of 5 mg/mL, 10 mg/mL, 15 mg/mL, and 20 mg/mL, respectively, and other ROIs with mixed materials, such as muscle, lung tissue, and bone; whereas the suitcase phantom includes three ROIs 0–2 of single-element calibration materials, i.e., carbon, aluminum, and calcium, and four more ROIs 3–6 of mixed materials, corresponding to water, ANFO (Ammonium Nitrate and Fuel Oil [11]), teflon, and PVC, respectively.

In DECT, basis images may be selected according to the task considered. For the chest phantom, to estimate iodine concentrations, we select material-based basis images of water and iodine concentration of 20 mg/mL, with the corresponding $\mu_{km}$’s obtained from the NIST database [24]. For the suitcase phantom, in order to estimate effective atomic numbers, we select interaction-based basis images of the photoelectric effect (PE) and Compton scattering (KN) with $\mu_{km}$’s that are $1/E^{3}$, where *E* denotes X-ray energy, and obtained with the Klein–Nishina formula [1], respectively. The basis images and VMIs of the chest and suitcase phantoms are formed on image arrays of 200×256 and 150×256 square pixels of size 0.7 mm, as displayed in rows 1 and 2, respectively, in Figure 2.

### 2.4. Image Reconstruction Approach

In an attempt to compensate for the BH effect inherent in $g_{j}^{L}$ and $g_{j}^{H}$, we rewrite Equation (1) as

(3) $$\begin{array}{c} \begin{array}{cc} g_{j}^{L} & =-\ln\sum_{m}^{M}q_{jm}^{L}\exp\left(-\mu_{0m}l_{0j}-\mu_{1m}l_{1j}\right),\\ g_{j}^{H} & =-\ln\sum_{m}^{M}q_{jm}^{H}\exp\left(-\mu_{0m}l_{0j}-\mu_{1m}l_{1j}\right), \end{array} \end{array}$$

where $l_{kj}=\sum_{i}^{I}a_{ji}b_{ki}$, k=0 or 1, denotes the sinogram of basis image *k*, also referred to as basis data, which is independent of energy *m*. Therefore, applying the DDD algorithm [10] to Equation (3), we can obtain basis sinograms $l_{kj}$ from knowledge of $g_{j}^{L}$ and $g_{j}^{H}$ for each ray *j*. It has been shown empirically [25] that the DDD algorithm can recover accurately basis sinograms from $g_{j}^{L}$ and $g_{j}^{H}$. Using existing algorithms such as the FBP algorithm, one can reconstruct readily accurate basis images from full knowledge of basis sinograms $l_{kj}$ in a FAR or short scan. In the work, because knowledge of $l_{kj}$ can be available only over a SA or TOA of LARs, the FBP algorithm yield basis images of significant artifacts. We thus develop and tailor the DTV algorithm to reconstruct basis images with minimized LAR artifacts from knowledge of $l_{kj}$’s available only over a SA or TOA of LARs.

Using vectors $b_{k}$ and $L_{k}$ (k=0 or 1) of sizes *I* and *J*, respectively, to denote basis images and their sinograms with elements $b_{ki}$ and $l_{kj}$ in concatenated forms, we formulate the reconstruction problem of basis images from their sinograms as a convex optimization problem

(4) $$\begin{array}{c} \begin{array}{cc} b_{k}^{★} & =\underset{b_{k}}{\mathrm{argmin}}\;\;\frac{1}{2}{\left\|L_{k}-A\;b_{k}\right\|}_{2}^{2}\\ \;s.t.\;\; & \left|\right|D_{x}b_{k}{\left|\right|}_{1}\leq t_{kx},\;\;||D_{y}b_{k}{\left|\right|}_{1}\leq t_{ky},\;\;\mathrm{and}\;\;b_{ki}\geq 0, \end{array} \end{array}$$

where matrix A of size J×I denotes the discrete fan-beam X-ray transform with element $a_{ji}$; ${\left\|\cdot \right\|}_{2}$ the $\ell_{2}$-norm of a vector; and $\left|\right|D_{x}b_{k}{\left|\right|}_{1}$ and $\left|\right|D_{y}b_{k}{\left|\right|}_{1}$ are the image’s directional total variations (DTVs) [5] of the basis image $b_{k}$ along the *x*- and *y*-axis, respectively.

The DTV algorithm used to reconstruct basis images from knowledge of the basis sinograms in DECT through solving Equation (4) shares the same general structure as that of the algorithm for image reconstruction from LAR data in conventional SECT [5]. The pseudo-code is thus summarized in Appendix A for clarity.

### 2.5. Visual Inspection and Quantitative Analysis of Images

As VMIs are of visualization interest in DECT, we first obtain VMIs at energy levels of interest from basis images reconstructed by using Equation (2) and then visually inspect LAR artifacts in the VMIs. Additionally, two quantitative metrics, normalized root-mean-square error (nRMSE) and Pearson correlation coefficient (PCC) [5,26,27] are calculated. Metric nRMSE evaluates quantitative difference, while metric PCC assesses visual correlation, between a VMI obtained from LAR data and a reference image obtained from FAR data. In particular, higher PCCs suggest better visual correlation between the VMI and its reference image. The VMI and its reference are identical when PCC → 1 and nRMSE → 0.

In the chest phantom study, we seek to estimate iodine-contrast concentration within ROIs 1–4 shown in the basis images in row 1 of Figure 2. Using the estimated basis image of 20-mg/mL iodine-contrast agent, we estimate the concentration of iodine-contrast agent within ROIs 1–4 with a linear fitting [6]. Constants in the linear relationship are determined by using pixel values within iodine-contrast ROIs 1–4 in the reference image of the chest phantom obtained from the FAR data by use of the two-step method, and fitting into the corresponding known concentrations. In the work, the calibrated slope and intercept of the linear fitting were computed as 19.3 mg/mL and −0.0074 mg/mL. In general, the linear fitting, as compared to the default setting of 20 and 0 as slope and intercept, yields more accurate estimation of the iodine concentration, because the mean pixel values within ROI 0 in the 20-mg/mL iodine basis image could be non-zero. This occurs as a result of the incomplete basis set in the material decomposition model by using 2 materials. On the other hand, in the study involving the suitcase phantom, we seek to estimate the effective atomic number of materials [11]. As the basis images are estimated as PE and KN components, their ratios are used in an affine transform with the effective atomic number in the log-log domain [6]. The effective atomic numbers are then computed for ROIs 3–6 of the suitcase phantom, as shown in row 2 of Figure 2. Constants in the affine transformation are determined by using the pixel values within single-element ROIs 0–2 in the reference image of the suitcase phantom obtained from the FAR data by use of the two-step method, and fitting into the corresponding known atomic numbers.

## 3. Results

### 3.1. Numerical Study Design and Data Generation

In our numerical studies with noiseless and noisy LAR data, the TASMIC model [28] was used for generating filtered tube spectra of given low- and high-kVps. Taking into account the detector’s energy-integrating response, we then obtain $q_{jm}^{L}$ and $q_{jm}^{H}$ by multiplying the filtered tube spectra with corresponding X-ray energies *E*. For both phantoms, the low- and high-kVp spectra are set at 80 and 140 kVp, with a 5-mm Al filter used in both.

For each of the chest or suitcase phantom in an SA or TOA scan described in Section 2.1 above, basis sinograms $l_{kj}$ are first generated from basis images shown in Figure 2, and noiseless low- and high-kVp data $g_{j}^{L}$ and $g_{j}^{H}$ can be generated subsequently by use of Equation (3) with $l_{kj}$, and knowledge of $\mu_{km}$, $q_{jm}^{L}$, and $q_{jm}^{H}$ determined. The aims of the noiseless data study are (1) to verify that the two-step method, including the DDD and DTV algorithms, can recover numerically accurate basis images and VMIs first from FAR-scan data and (2) to use the two-step method verified to explore empirically its performance upper bound , i.e., the performance in the best case scenario without any inconsistencies, such as noise and decomposition error, in the data, as a function of LARs for yielding accurate reconstruction of VMIs and physical quantity estimation in DECT with LAR scans.

Using noiseless data as the means of the Poisson noise model, we obtain noisy data containing Poisson noise. For both chest and suitcase phantoms, Table 1 shows the noise-equivalent quanta’s (NEQs) of each detector bin for the SA or TOA scans studied, which are determined such that the means in SA or TOA scans studied have a fixed total number of quanta of ∼$6.9\times 10^{9}$ in an air scan, amounting to 75% of that in a FAR scan with 360 projection views, 512 detector bins, and ∼$5\times 10^{4}$ NEQs per detector bin [23]. The purpose of the noisy data study is to yield some preliminary insights into the reconstruction robustness of the two-step method. Clearly, its reconstruction accuracy depends not only on the LAR extent but also on the characteristics and level of data noise. No additional data or image processing is applied in the study with noisy data, although such processing may improve the quality of VMI visualization and physical quantity estimation.

Constraint parameters $t_{kx}$ and $t_{ky}$ have an impact on image reconstruction by defining the feasible solution set of Equation (4). In the study below with consistent noiseless data, the DTV values of the phantom basis images in Figure 2 are selected as the values of parameters $t_{kx}$ and $t_{ky}$, in order to form the tightest feasible solution set that still contain the desired solution (i.e., the truth basis images in this case). In the study with noisy data, the values of parameters $t_{kx}$ and $t_{ky}$ are selected in terms of visual evaluation of reconstructed VMIs with minimum artifacts [5,6]. In general, parameter selection is accomplished through surveying the parameter space within relevant ranges and optimizing a well-defined image-quality metric, e.g., image visualization for artifact reduction or quantitative estimation of iodine-contrast concentration, for studies with inconsistent data, including those with real data where the truth images are not available. In our experience, $t_{kx}$ and $t_{ky}$ selected in the noisy data studies are generally smaller than those in the corresponding noiseless data studies. In the work, the $t_{kx}$ values selected are in general smaller than $t_{ky}$ in the SA scans, so as to suppress horizontal streaks along the *y*-axis, while both $t_{kx}$ and $t_{ky}$ selected in the TOA scans are slightly larger than those in the SA scans, as the improved conditioning of the system matrix leads to fewer artifacts overall. Basis images are also reconstructed from $L_{k}$ estimated by using the FBP algorithm with a Hanning kernel and a cutoff frequency at 0.5, which are then combined into the VMIs with Equation (2). The FBP algorithm is used only for demonstrating the LAR artifacts associated with the phantoms and data conditions in the work.

### 3.2. Image Reconstruction of the Chest Phantom

#### 3.2.1. Verification Study with the Chest Phantom

**Figure 3:**
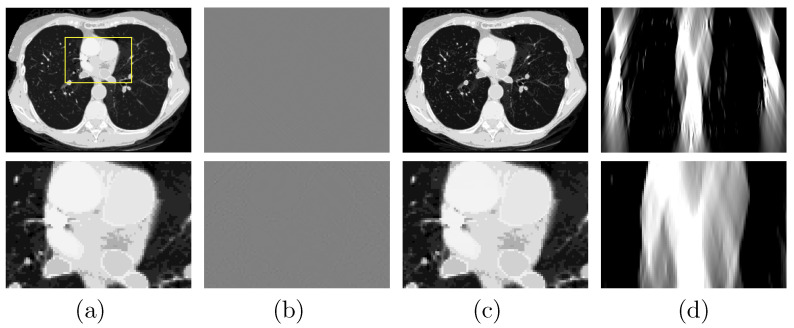
Row 1: (**a**) VMI of the chest phantom at 100 keV obtained with the two-step method from FAR data, (**b**) difference between the VMI in (**a**) and its truth in Figure 2c, and VMIs at 100 keV obtained with (**c**) the two-step method and (**d**) the FBP algorithm from noiseless data acquired over a SA of LAR $30^{\circ }$; Row 2: zoomed-in views of their corresponding images in row 1. The zoomed-in area is enclosed by the rectangular box depicted in the VMI in (**a**). Display windows [0, 0.22] cm${}^{-1}$ for columns (**a**,**c**,**d**), and [$-10^{-4},10^{-4}$] cm${}^{-1}$ for column (**b**).

A study is performed to first verify that (1) the DDD algorithm can invert the non-linear model in Equation (3) and numerically accurately recover the basis sinogram $l_{kj}$ from noiseless low- and high-kVp data of the chest phantom acquired with the FAR scan of $360^{\circ }$; and (2) the DTV algorithm developed and tailored can reconstruct numerically accurate basis images and VMIs from noiseless basis sinogram. In Figure 3a, we display the VMI at 100 keV [29], along with a zoomed-in view, and show in Figure 3b their differences from the truth counterparts in Figure 2c (top row). The result confirms that the two-step method can yield accurate reconstructions from noiseless FAR data. In an attempt to demonstrate possible LAR artifacts associated with the phantom, we apply both the DTV and FBP algorithms to reconstructing images from noiseless basis sinogram acquired with a SA scan of LAR $\alpha_{\tau }=30^{\circ }$, and display them in Figure 3c,d, respectively. It can be observed that the two-step method can significantly reduce the LAR artifacts observed in the FBP image.

#### 3.2.2. Image Reconstruction from Noiseless Data Acquired with SA and TOA Scans of LARs

**Figure 4:**
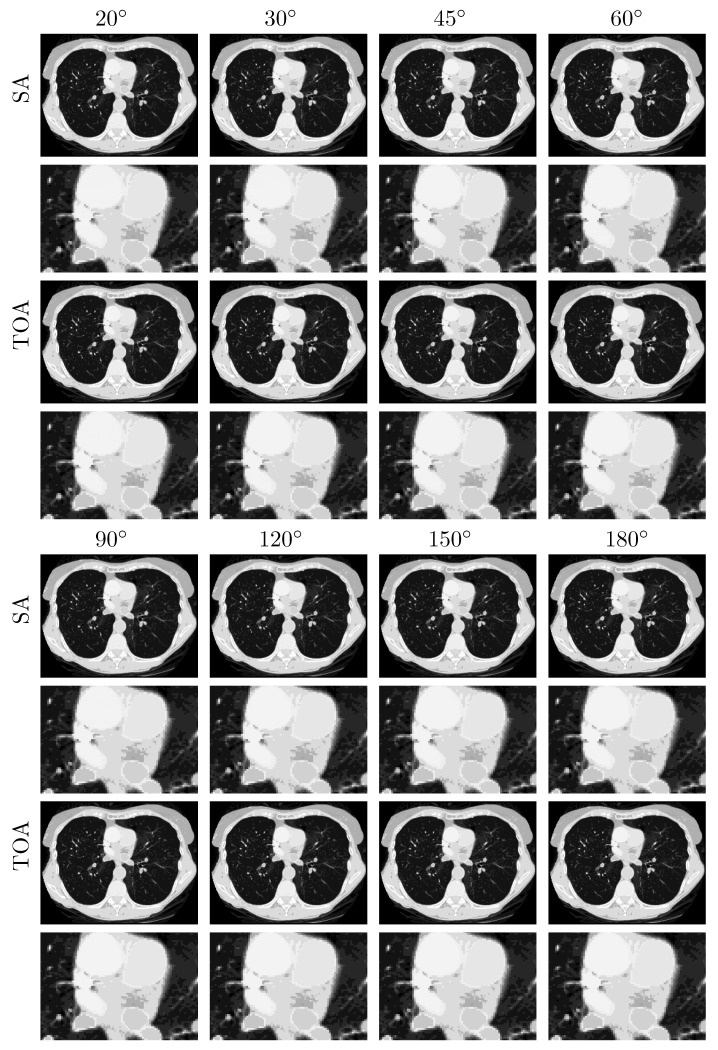
VMIs (rows 1, 3, 5, and 7), along with their respective zoomed-in views (rows 2, 4, 6, and 8), of the chest phantom at 100 keV obtained from noiseless data over SAs (rows 1&2 and 5&6) and TOAs (rows 3&4 and 7&8) of LAR $20^{\circ }$, $30^{\circ }$, $45^{\circ }$, $60^{\circ }$, $90^{\circ }$, $120^{\circ }$, $150^{\circ }$, and $180^{\circ }$, respectively, by use of the two-step method. Display window: [0, 0.22] cm${}^{-1}$.

We subsequently apply the two-step method verified to reconstructing basis images of water and iodine from noiseless data of the chest phantom collected in SA or TOA scans of $\alpha_{\tau }=20^{\circ },30^{\circ },45^{\circ },60^{\circ },90^{\circ },120^{\circ }$, $150^{\circ }$, and $180^{\circ }$. In Figure 4, we display VMIs at 100 keV, along with their zoomed-in views within the ROI, reconstructed for the SA (rows 1&2 and 5&6) and TOA (rows 3&4 and 7&8) scans. It can be observed that the two-step method yields visually comparable images for these scans of LARs, revealing quantitatively possible performance upper bounds of the two-step method in accurate image reconstruction, i.e., numerically accurately inverting Equation (3), for SA and TOA scans of LARs studied in the work.

**Figure 5:**
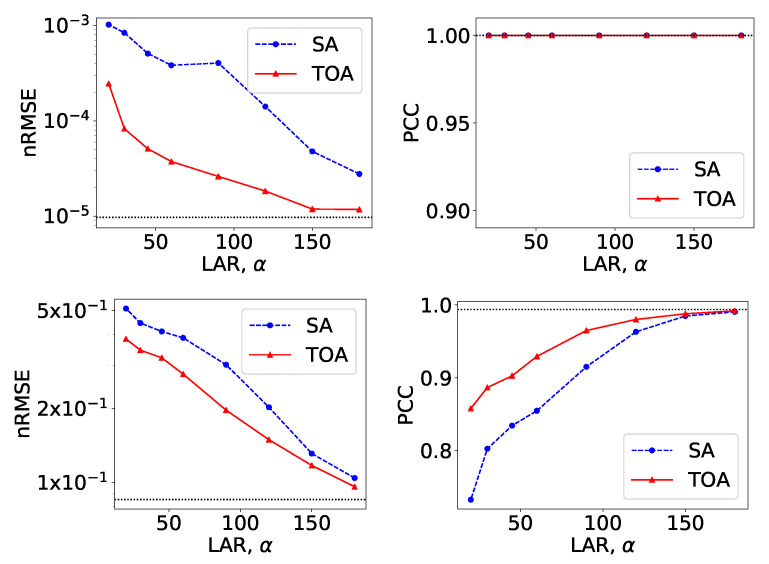
Metrics nRMSE and PCC, computed over VMIs of the chest phantom from noiseless data in Figure 4 (row 1) and those from noisy data in Figure 7 (row 2) as functions of LARs $\alpha_{\tau }$ for SA (blue, dashed) and TOA (red, solid) scans. The horizontal lines (black, dotted) indicate the reference values from FAR data of $360^{\circ }$.

**Figure 6:**
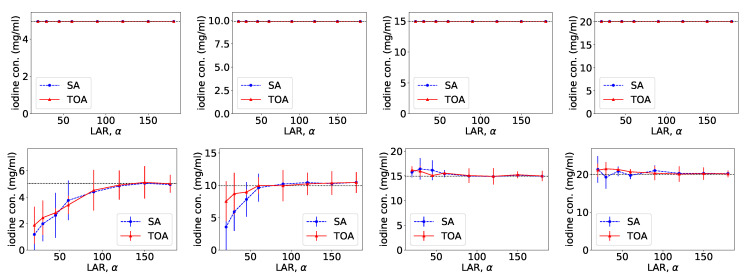
Iodine concentrations, along with their respective error bars, in ROIs 1–4 (from left to right) within the chest phantom, as functions of LARs $\alpha_{\tau }$ for SA (blue, dashed) and TOA (red, solid) scans, estimated from basis images reconstructed from noiseless (row 1) and noisy (row 2) data by use of the two-step method.

From VMIs in Figure 4, we compute nRMSEs and PCCs, which are displayed in row 1 of Figure 5. Using the method described in Section 2.5, we also estimate iodine concentrations in ROIs 1–4 indicated in the top row of Figure 2c, and plot them as functions of LARs in row 1 of Figure 6. These results reveal that, from the chest phantom noiseless data collected over the range of LARs as low as $20^{\circ }$, the two-step method can yield VMIs visually and quantitatively close to the reference VMIs from FAR data of $360^{\circ }$ in terms of PCC and estimated iodine concentrations. Regarding metric nRMSE, it increases as LAR decreases, mainly due to the increasing null spaces present in the system matrices of the LAR scans, while TOA scans can lower nRMSE by an order of magnitude especially for small LARs as compared to SAs of the same LAR.

#### 3.2.3. Image Reconstruction from Noisy Data Acquired with SA and TOA Scans of LARs

**Figure 7:**
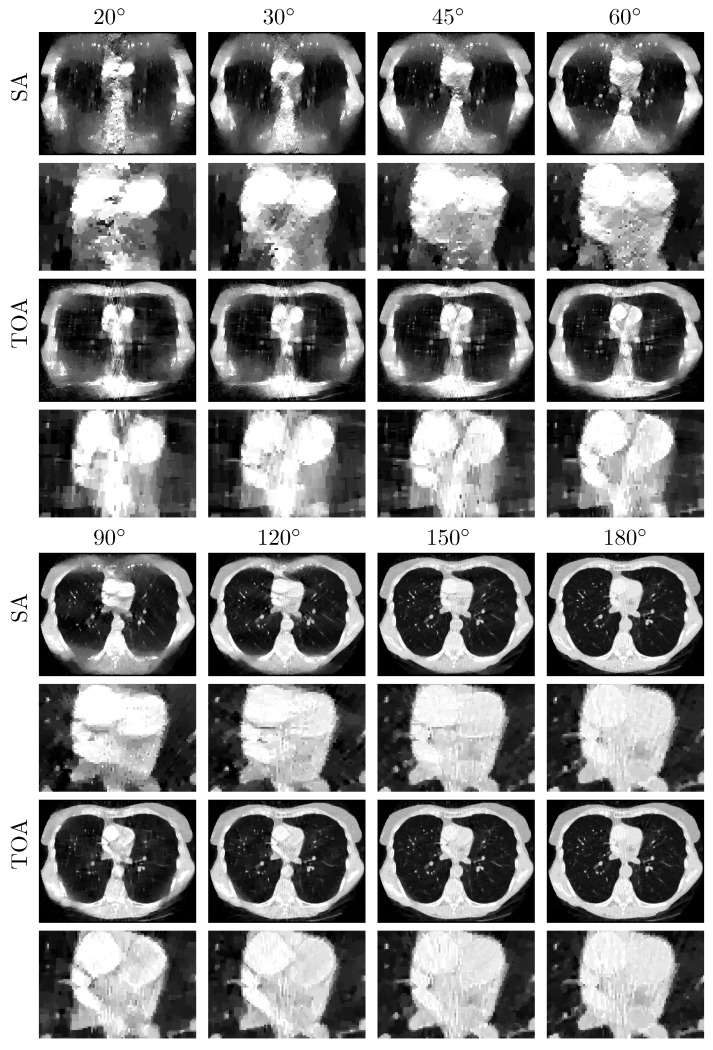
VMIs (rows 1, 3, 5, and 7), along with their respective zoomed-in views (rows 2, 4, 6, and 8), of the chest phantom at 100 keV obtained from noisy data over SAs (rows 1&2 and 5&6) and TOAs (rows 3&4 and 7&8) of LAR $20^{\circ }$, $30^{\circ }$, $45^{\circ }$, $60^{\circ }$, $90^{\circ }$, $120^{\circ }$, $150^{\circ }$, and $180^{\circ }$, respectively, by use of the two-step method. Display window: [0, 0.22] cm${}^{-1}$.

We repeat the study by applying the DTV algorithm to noisy data of the chest phantom for SA and TOA scans considered in the noiseless study above. In Figure 7, we display VMIs at 100 keV, along with their zoomed-in views. For the noise levels considered, it can be observed that (1) LAR artifacts can be amplified by noise, (2) LAR artifacts are reduced substantially in VMIs for SA and TOA scans of LARs $\alpha_{\tau }\geq 120^{\circ }$, and (3) TOA scans can more effectively suppress LAR artifacts than SA scans for the chest phantom and noise level studied in the work. Such observations may provide insights into the design of practical procedures for image reconstruction from LAR data that contain additional inconsistencies. We note that no processing is applied to the data or images reconstructed in our study.

Similar to the noiseless-data study, we compute nRMSEs and PCCs from VMIs in Figure 7, and plot them as functions of LARs in row 2 of Figure 5. We also estimate iodine concentrations and plot them as functions of LAR in row 2 of Figure 6. In the noisy-data study, error bars, i.e., standard deviations, are calculated over the chest-phantom ROIs indicated in Figure 2c, and they are plotted in row 2 of Figure 6. The horizontal lines (black, dotted) indicate the reference values from FAR data of $360^{\circ }$. Quantitative results of PCC appear consistent with the visual inspection, suggesting that VMI images for $\alpha_{\tau }\geq 120^{\circ }$ in SA and $\alpha_{\tau }\geq 90^{\circ }$ in TOA scans visually resemble the reference VMI obtained from noisy FAR data, and the degree of resemblance drops understandably as LAR decreases. The estimation accuracy of iodine concentration for $\alpha_{\tau }\geq 90^{\circ }$ remains comparable to those obtained from the reference images reconstructed from noisy FAR data.

### 3.3. Image Reconstruction of the Suitcase Phantom

**Figure 8:**
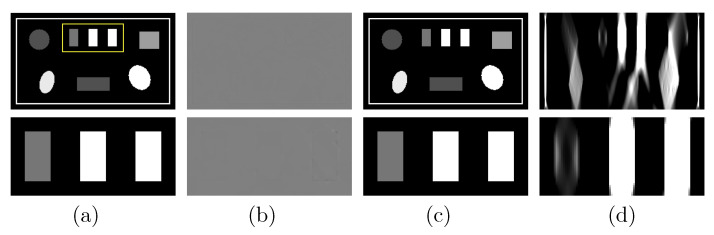
Row 1: (**a**) VMI of the suitcase phantom at 40 keV obtained with the two-step method from FAR data, (**b**) difference between the VMI in (**a**) and its truth in Figure 2c, VMIs at 40 keV obtained with (**c**) the two-step method and (**d**) the FBP algorithm from data acquired over a SA of LAR $30^{\circ }$; and row 2: zoomed-in views of their corresponding images in row 1. The zoomed-in area is enclosed by the rectangular box depicted in the VMI in (**a**). Display windows [0.1, 0.65] cm${}^{-1}$ for columns (**a**,**c**,**d**), and [$-10^{-4},10^{-4}$] cm${}^{-1}$ for column (**b**).

Next, we repeat the studies in Section 3.2 with the suitcase phantom. We show in Figure 8a the VMI and its zoomed-in view reconstructed from FAR data and in Figure 8b their differences from the truth counterparts in Figure 2c (bottom row). The result again confirms the reconstruction accuracy of the two-step method using the suitcase phantom, which is of different complexity and structure to the chest phantom. To reveal the LAR artifacts associated with the suitcase phantom, we apply the DTV and FBP algorithms to reconstruct images from noiseless basis sinogram over an SA of $\alpha_{\tau }=30^{\circ }$ and display them in Figure 8c,d, respectively. It can be observed that the LAR artifacts in the FBP image are almost eliminated in the image reconstructed by use of the two-step method.

#### 3.3.1. Image Reconstruction from Noiseless Data Acquired with SA and TOA Scans of LARs

**Figure 9:**
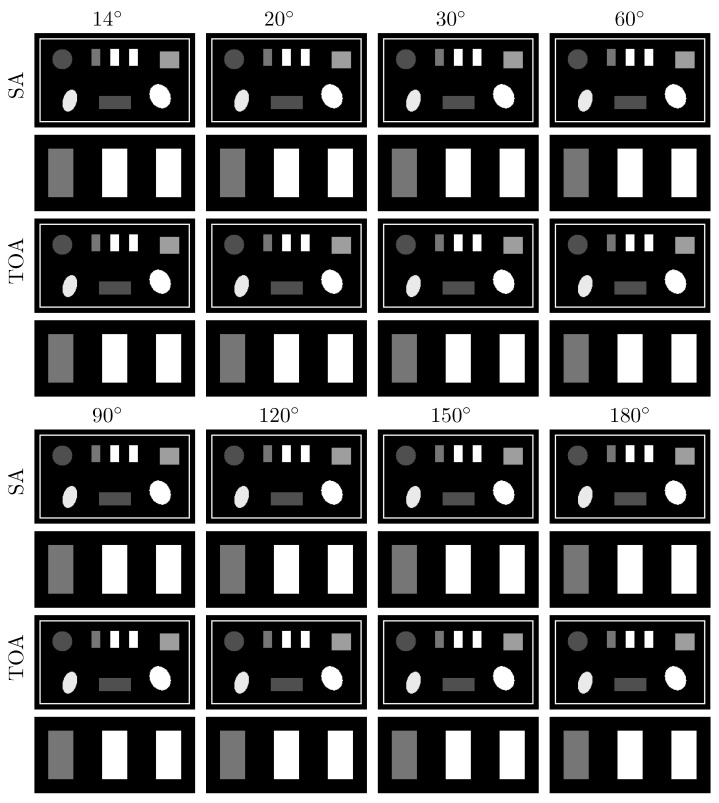
VMIs (rows 1, 3, 5, and 7), along with their respective zoomed-in views (rows 2, 4, 6, and 8), of the suitcase phantom at 40 keV obtained from noiseless data acquired over SAs (rows 1&2 and 5&6) and TOAs (rows 3&4 and 7&8) of LAR $14^{\circ }$, $20^{\circ }$, $30^{\circ }$, $60^{\circ }$, $90^{\circ }$, $120^{\circ }$, $150^{\circ }$, and $180^{\circ }$, respectively, by use of the two-step method. Display window: [0.1, 0.65] cm${}^{-1}$.

Next, we apply the algorithm verified to reconstructing basis images of PE and KN from noiseless data of the suitcase phantom collected in SA or TOA scans of $\alpha_{\tau }=14^{\circ },20^{\circ },30^{\circ },60^{\circ },90^{\circ },120^{\circ }$, $150^{\circ }$, and $180^{\circ }$. The lowest LAR studied for the suitcase phantom, $14^{\circ }$, is smaller than that for the chest phantom, $20^{\circ }$. In Figure 9, we display the VMIs at 40 keV, along with their zoomed-in views, reconstructed from data collected with SA (rows 1&2 and 5&6) and TOA (rows 3&4 and 7&8) scans. It can be observed that the two-step method yields almost visually identical images for these LARs, revealing possible performance upper bounds of the method in numerically accurately inverting Equation (3) for scans with SA and TOA of LARs.

**Figure 10:**
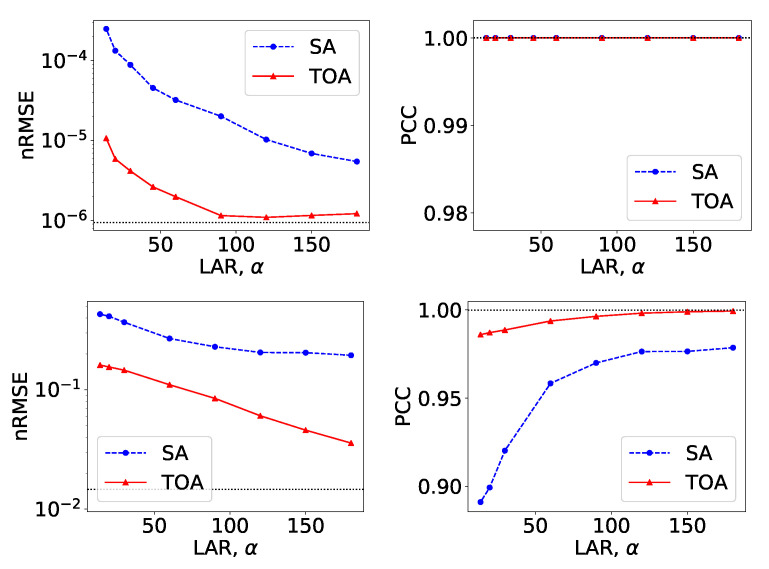
Metrics nRMSE and PCC, computed over VMIs of the suitcase phantom from noiseless data in Figure 9 (row 1) and those from noisy data in Figure 12 (row 2) as functions of LARs $\alpha_{\tau }$ for SA (blue, dashed) and TOA (red, solid) scans. The horizontal lines (black, dotted) indicate the reference values from FAR data of $360^{\circ }$.

**Figure 11:**
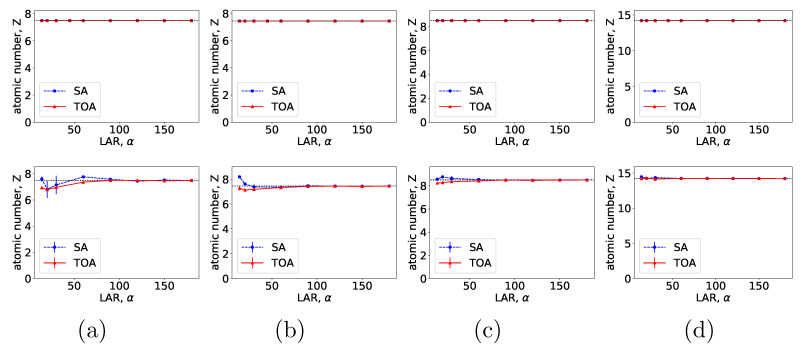
Effective atomic numbers of (**a**) water, (**b**) ANFO, (**c**) Teflon, and (**d**) PVC, along their respective error bars, within the suitcase phantom estimated as functions of LAR $\alpha_{\tau }$ for SA (blue, dashed) and TOA (red, solid) scans, computed from basis images reconstructed from noiseless (row 1) and noisy (row 2) data by use of the two-step method.

From VMIs in Figure 9, we compute nRMSEs and PCCs, and display them in row 1 of Figure 10. Using the method described in Section 2.5, we also estimate effective atomic numbers in ROIs 3–6 indicated in bottom row of Figure 2c, and plot them as functions of LARs in row 1 of Figure 11. These results reveal that, from the suitcase phantom noiseless data collected over the range of LARs as low as $14^{\circ }$, the two-step method can yield VMIs visually and quantitatively close to the reference VMIs from FAR data of $360^{\circ }$ in terms of PCC and estimated effective atomic numbers. With regard to metric nRMSE, it increases as LAR decreases, largely due to the increasing null spaces in the system matrices of the LAR scans, while TOA scans can lower nRMSE by an order of magnitude especially for small LARs as compared to SAs of the same LAR.

#### 3.3.2. Image Reconstruction from Noisy Data Acquired with SA and TOA Scans of LARs

**Figure 12:**
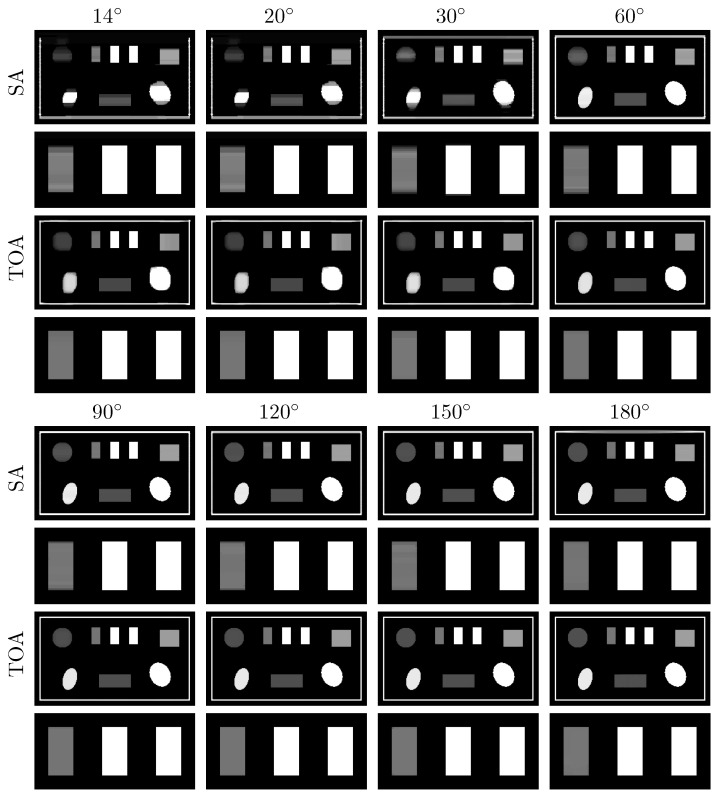
VMIs (rows 1, 3, 5, and 7), along with their respective zoomed-in views (rows 2, 4, 6, and 8), of the suitcase phantom at 40 keV obtained from noisy data acquired over SAs (rows 1&2 and 5&6) and TOAs (rows 3&4 and 7&8) of LAR $14^{\circ }$, $20^{\circ }$, $30^{\circ }$, $60^{\circ }$, $90^{\circ }$, $120^{\circ }$, $150^{\circ }$, and $180^{\circ }$, respectively, by use of the two-step method. Display window: [0.1, 0.65] cm${}^{-1}$.

We apply the two-step method to reconstructing images from noisy data of the suitcase phantom collected over the same LARs in SA and TOA. In Figure 12, we display the VMIs at 40 keV, along with their zoomed-in views. For the suitcase phantom, LAR artifacts are substantially reduced in VMIs from data collected over $\alpha_{\tau }\geq 90^{\circ }$ in SA and $\alpha_{\tau }\geq 60^{\circ }$ in TOA scans. Similar to the chest phantom results, TOA configurations can more effectively suppress LAR artifacts than SA ones, especially recovering the distorted edges around the circular and elliptical disks, for the suitcase phantom under noise level studied in the work.

We compute nRMSEs and PCCs from VMIs in Figure 12, and plot them as functions of LARs in row 2 of Figure 10. We also estimate effective atomic numbers and plot them as functions of LAR in row 2 of Figure 11. In the noisy-data study, error bars, i.e., standard deviations, are calculated over the suitcase-phantom ROIs indicated in Figure 2c, and they are plotted in row 2 of Figure 11. The quantitative results suggest that VMI images visually resemble the reference VMI from FAR data for noisy LAR data collected over $\alpha_{\tau }\geq 60^{\circ }$ in SA and $\alpha_{\tau }\geq 14^{\circ }$ in TOA scans, and the resemblance decreases understandably as LAR decreases and that the estimation accuracy from noisy LAR data collected over $\alpha_{\tau }\geq 60^{\circ }$ is comparable to those from the FAR data.

## 4. Discussion

In this work, we have investigated and developed a two-step method for image reconstruction from low- and high-kVp data collected with SA and TOA scans of LARs in DECT. The method combines the DDD and DTV algorithms to effectively compensate for both BH and LAR artifacts, yielding accurate VMIs and physical-quantity estimation. For the study conditions such as phantoms and noise levels considered, visual inspection of VMIs at energies of interest indicates that the method can yield from noiseless LAR data VMIs that are visually comparable to the reference VMI from FAR data, and from noisy LAR data VMIs with reduced BH and LAR artifacts; and quantitative observations can be made that the accurate estimation of physical quantities such as iodine concentrations and effective atomic numbers can be obtained for noiseless data of LAR as low as $20^{\circ }$ and for noisy data of LAR as low as $60^{\circ }$. For the SA and TOA scans of the same total angular range studied, the latter appear to yield more accurate images and estimations of physical quantities than the former, due to the improved conditioning of the system matrix.

We used two distinct phantoms, i.e., chest and suitcase phantoms, of varying complexity levels and structures of different application interest. The chest phantom contains lung tissue, airways, and blood vessels within the pulmonary anatomy, while the suitcase phantom contains various materials of interest in baggage screening. Results of the numerical study indicate that the effectiveness of the two-step method, like any other algorithm, is understandably dependent on the anatomic complexity of an object imaged with varying contrast and spatial resolution. Results from the suitcase phantom are less impacted, in terms of image artifacts and quantitative accuracy of the estimated physical quantities, by the decreasing LAR than the chest phantom, possibly due to its structure and the noise levels in the data. We have studied additional phantoms of different anatomies, and corroborative observations can be made.

In the work, we have investigated the DTV algorithm for numerically accurately solving the optimization program in Equation (4) with DTV constraints. Additionally, we have conducted noisy data studies to provide some preliminary insights into the stability of the two-step method in the presence of data inconsistencies. While a fixed total number of quanta is used for Poisson noise simulation, the visualization of VMIs and estimation accuracy of physical quantities obtained can be dependent on the noise levels and characteristics of different applications. In addition, other sources of inconsistency, such as metal, scatter, imperfect spectra, low- and high-kVp X-ray mismatch, and decomposition error, may also impact the reconstruction quality and estimation accuracy. Blooming artifacts usually stem from highly attenuating materials present in the patient, such as metal implants and calcification plaques. While it is important to investigate the effectiveness of the two-step method in studies containing these physical effects, such an investigation nevertheless is beyond the scope of this work, and the proposed method may be used as the basis for future investigative efforts that focus on correcting other physical factors in DECT with LAR data.

The studies and results in this work may provide insights into the possible development for practical approaches to reducing radiation dose and scanning time and to avoiding collision between the moving gantry of the scanner and the imaged object in clinical and industrial applications. One limitation of the proposed two-step method is the requirement of completely overlapping arcs of low- and high-kVp scans, imposed by the data-domain decomposition step. This can be avoided by performing the image-domain decomposition in a two-step method [30]; however, linear data models are usually assumed and the non-linear BH effect is not explicitly corrected for, which may impact the quantitative accuracy of the reconstruction. On the other hand, one-step methods [25] may accommodate LAR scanning configurations with partially or non-overlapping arcs of low- and high-kVp scans, while using the non-linear data model and correcting for the BH effect. Therefore, future investigations will include studies on one-step methods for DECT reconstruction with LAR data. It is worthy of a separate, comprehensive investigation, since existing studies on one-step methods focus largely on full- or short-angular-range scans and leverage image constraints, such as TV, not specifically designed for LAR data [15,25].

## 5. Conclusions

In this work, we investigated and developed a two-step method to reconstruct images accurately from low- and high-kVp LAR data by correcting for both BH and LAR effects in DECT. Numerical studies conducted reveal that the two-step method can yield VMIs with reduced BH and LAR artifacts, and estimation of physical quantities with improved accuracy, and that for SA and TOA scans with identical total LARs, the latter generally yields more accurate image reconstruction and physical-quantity estimation than the former. Results and knowledge acquired in the work on accurate image reconstruction in LAR DECT may give rise to further understanding and insights into the practical design of LAR scan configurations and reconstruction procedures for DECT applications. Future works will investigate the impact of additional inconsistencies and the one-step method for accommodating non-overlapping scans in DECT with LAR data.

## Figures and Tables

**Table 1:** NEQs per detector bin in air scans of either the low- or high-kVp scans for the chest and suitcase phantoms with LARs ranging from $14^{\circ }$ to $180^{\circ }$, as well as with the FAR of $360^{\circ }$.

LAR	$14^{\circ }$	$20^{\circ }$	$30^{\circ }$	$45^{\circ }$	$60^{\circ }$
NEQ	$9.64\times 10^{5}$	$6.75\times 10^{5}$	$4.5\times 10^{5}$	$3\times 10^{5}$	$2.25\times 10^{5}$
LAR	$90^{\circ }$	$120^{\circ }$	$150^{\circ }$	$180^{\circ }$	$360^{\circ }$
NEQ	$1.5\times 10^{5}$	$1.125\times 10^{5}$	$9\times 10^{4}$	$7.5\times 10^{4}$	$3.75\times 10^{4}$

## Data Availability

The data presented in this study are available on request from the corresponding author.

## Abbreviations

    The following abbreviations are used in this manuscript:

DECT	Dual-energy computed tomography
LAR	Limited angular range
BH	Beam hardening
DDD	Data-domain decomposition
DTV	Directional total variation
VMI	Virtual monochromatic image
SA	Single-arc
TOA	Two-orthogonal-arc
FAR	Full angular range
nRMSE	Normalized root-mean-square error
PCC	Pearson correlation coefficient
PE	Photoelectric
KN	Klein–Nishina
NEQ	Noise-equivalent quanta

## Appendix A. Pseudo-Code of the DTV Algorithm

**Algorithm A1 **Pseudo-code of the DTV algorithm for solving Equation (4)
1: INPUT: $L_{k}$, $t_{kx}$, $t_{ky}$, A, ρ 2: $L\leftarrow {\left||K|\right|}_{2}$, τ←ρ/L, σ←1/(ρL), $\nu_{1}\leftarrow {\left||A|\right|}_{2}/\left|\right|D_{x}{\left|\right|}_{2}$, $\nu_{2}\leftarrow {\left||A|\right|}_{2}/\left|\right|D_{y}{\left|\right|}_{2}$, $\mu \leftarrow {\left||A|\right|}_{2}/{\left||I|\right|}_{2}$ 3: n←0 4: INITIALIZE: $b^{\left(0\right)}$, $w^{\left(0\right)}$, $p^{\left(0\right)}$, $q^{\left(0\right)}$, and $t^{\left(0\right)}$ to zero 5: ${\bar{b}}^{\left(0\right)}\leftarrow b^{\left(0\right)}$ 6: **repeat** 7: $w^{\left(n+1\right)}=\left(w^{\left(n\right)}+\sigma \left(A{\bar{b}}^{\left(n\right)}-L\right)\right)/\left(1+\sigma \right)$ 8: $p^{\prime (n)}=p^{\left(n\right)}+\sigma \nu_{1}D_{x}{\bar{b}}^{\left(n\right)}\;q^{\prime (n)}=q^{\left(n\right)}+\sigma \nu_{2}D_{y}{\bar{b}}^{\left(n\right)}$ 9: $p^{\left(n+1\right)}=p^{\prime (n)}-\sigma \frac{p^{\prime (n)}}{|p^{\prime (n)}|}\ell_{1}{\mathrm{ball}}_{\nu_{1}t_{kx}}\left(\frac{|p^{\prime (n)}|}{\sigma }\right)\;q^{\left(n+1\right)}=q^{\prime (n)}-\sigma \frac{q^{\prime (n)}}{|q^{\prime (n)}|}\ell_{1}{\mathrm{ball}}_{\nu_{2}t_{ky}}\left(\frac{|q^{\prime (n)}|}{\sigma }\right)$ 10: $t^{\left(n+1\right)}=\mathrm{neg}\left(t^{\left(n\right)}+\sigma \mu {\bar{b}}^{\left(n\right)}\right)$ 11: $b^{\left(n+1\right)}=b^{\left(n\right)}-\tau \left(A^{⊤}w^{\left(n+1\right)}+\nu_{1}D_{x}^{⊤}p^{\left(n+1\right)}+\nu_{2}D_{y}^{⊤}q^{\left(n+1\right)}+\mu t^{\left(n+1\right)}\right)$ 12: ${\bar{b}}^{\left(n+1\right)}=2b^{\left(n+1\right)}-b^{\left(n\right)}$ 13: n←n+1 14: **until** the convergence conditions are satisfied 15: OUTPUT: $b^{\left(n\right)}$ as the estimate of $b_{k}$

In the pseudo-code of the derived algorithm instance, the definitions of the auxiliary variables, including matrices K and I and vectors $w^{\left(n\right)}$, $p^{\prime (n)}$, $q^{\prime (n)}$, $p^{\left(n\right)}$, $q^{\left(n\right)}$, and $t^{\left(n\right)}$, and operators, including $\left|\right|\cdot {\left|\right|}_{2}$, neg(·), $\ell_{1}{\mathrm{ball}}_{\beta }\left(\cdot \right)$, and $|q^{\prime (n)}|$, are intentionally kept consistent with those used in Ref. [6]. In each reconstruction from a set of basis data in an SA and TOA scan, the DTV algorithm reconstructs the basis images through solving Equation (4) until the convergence conditions described in Ref. [5] are satisfied numerically.
